# Racial and ethnic differences in presentation and postoperative recovery after transsphenoidal surgery for cushing disease

**DOI:** 10.1007/s11060-026-05741-x

**Published:** 2026-08-06

**Authors:** Caleigh S. Roach, Vratko Himic, Richa Dahake, Khushi H. Shah, Victor M. Lu, Daniel Kreatsoulas, Arman Jahangiri, Carmen V. Villabona, Zoukaa B. Sargi, Ashish H. Shah, Carolina G. Benjamin, Ricardo J. Komotar, Michael E. Ivan

**Affiliations:** 1 Department of Neurological Surgery, University of Miami Miller School of Medicine, 1295 NW 14th Street, Suite 1700, Miami, FL 33125 USA; 2 Department of Endocrinology, University of Miami Miller School of Medicine, Miami, FL USA; 3 Department of Otolaryngology - Head & Neck Surgery, University of Miami Miller School of Medicine, Miami, FL USA

**Keywords:** Cushing’s disease, Transsphenoidal surgery, Pituitary adenomas, Healthcare disparities, Race and ethnicity, Biochemical remission

## Abstract

**Purpose:**

This study aimed to determine where along the surgical care pathway race- and ethnicity-associated differences emerge after primary transsphenoidal surgery (TSS) for Cushing’s disease (CD).

**Methods:**

Single-center retrospective cohort of 104 adults undergoing primary TSS for pathologically confirmed CD (2013–2024): 44 White non-Hispanic (WnH), 44 Hispanic, and 16 Black. Preoperative phenotype, perioperative complications, biochemical remission, radiographic persistence, and reintervention were compared across groups using unweighted statistics (Kruskal-Wallis, chi-square or Fisher’s exact tests). Sensitivity analysis used inverse probability of treatment weighting (IPTW) with a multinomial propensity model; Black subgroup comparisons were considered exploratory.

**Results:**

Hispanic patients lived closer to the center (*p* = 0.003) and presented with milder disease. Black patients were younger (*p* = 0.003), with higher BMI (*p* = 0.033) and near-uniformly solid tumors (94%; *p* = 0.040). Postoperative arginine vasopressin deficiency was less frequent in Hispanic than WnH patients (25% vs. 55%; *p* = 0.005). Durable remission rates were comparable (WnH 79%, Hispanic 81%, Black 88%), though Black patients had higher day-1 cortisol and delayed remission on IPTW sensitivity, higher radiographic persistence (38% vs. 21%), and higher crude reintervention (25% vs. 4.5%; *p* = 0.036). On exploratory IPTW analysis, Hispanic patients also showed shorter reintervention-free survival despite favorable baseline features (IPTW HR 4.20; *p* = 0.036), a signal not apparent in unadjusted analysis.

**Conclusion:**

Race- and ethnicity-associated differences emerged mainly at baseline presentation and in postoperative trajectory, whereas durable biochemical remission converged across groups; Black subgroup analyses were limited by small sample size and residual imbalance after weighting. These hypothesis-generating findings support postoperative surveillance tailored to where differences emerge rather than to ultimate remission and require validation in larger multicenter cohorts.

**Supplementary Information:**

The online version contains supplementary material available at https://doi.org/10.1007/s11060-026-05741-x.

## Introduction

Racial and ethnic disparities in surgical care persist across neurosurgical populations, with differences in disease presentation, perioperative complications, readmission, and long-term outcomes [1–3]. These have been attributed to access, socioeconomic status, insurance coverage, and referral patterns [4], though whether race and ethnicity remain independently associated with outcomes after adjustment is unclear.

Cushing’s disease (CD) is a high-morbidity endocrine disorder requiring timely surgical treatment [5]. Primary transsphenoidal surgery (TSS) is first-line therapy [5, 6], and early postoperative cortisol dynamics predict durable biochemical remission (BR) [7, 8]. Perioperative endocrine complications, including arginine vasopressin deficiency (AVP-D) and anterior pituitary dysfunction, affect recovery [9–11]. Because CD outcomes depend on tumor resection, postoperative endocrine management, and longitudinal biochemical surveillance within specialty care, differences in presentation, access, and perioperative care may drive variation [12].

Prior studies have reported racial and socioeconomic differences in incidence, presentation, and access to high-volume pituitary centers [4, 13–16], but data on CD surgical outcomes remain limited and largely rely on administrative datasets without complete socioeconomic adjustment [15]. The relationship between race and ethnicity and postoperative cortisol dynamics, remission timing, radiographic disease control, and reintervention after controlling for neighborhood deprivation and geographic access remains poorly characterized.

Our institution serves a diverse catchment area, allowing evaluation of race- and ethnicity-associated differences within a single high-volume center [17]. We therefore structured this analysis around where along the surgical care pathway differences emerge. We evaluated racial and ethnic differences in perioperative outcomes, remission timing and phenotype, radiographic disease-free survival (DFS), and reintervention following primary TSS for pathologically confirmed CD, applying multinomial inverse probability of treatment weighting (IPTW) as a sensitivity analysis to evaluate whether observed associations persisted after balancing baseline demographic, comorbidity, and access-related covariates.

## Methods

### Study design and patient population

We conducted a retrospective cohort study of adults undergoing primary TSS for CD between 2013 and 2024. CD required pathologic confirmation of a corticotroph pituitary adenoma (ACTH immunoreactivity and/or TPIT expression) and preoperative biochemical evidence of ACTH-dependent hypercortisolism per Endocrine Society and Pituitary Society consensus guidelines [18, 19]: at least two abnormal first-line tests (1-mg dexamethasone suppression test (DST) cortisol > 1.8 µg/dL; two late-night salivary cortisols (LNSC) above institutional threshold; or 24-hour urinary free cortisol (UFC) above the upper limit of normal) with ACTH ≥ 20 pg/mL [18]. Silent corticotroph adenomas, prior pituitary surgery, non-adenomatous or ectopic ACTH-dependent hypercortisolism, and incomplete records were excluded. Race and ethnicity were self-reported; the analytic cohort included 104 patients (44 WnH reference, 44 Hispanic, 16 Black), with Black subgroup analyses (*n* = 16) considered exploratory. Institutional Review Board approval was obtained (IRB# 20160437) with waiver of informed consent.

### Data collection

Clinical, socioeconomic, radiographic, operative, and endocrine data were extracted from the electronic record. Insurance was categorized as private, Medicare/Medicaid, or uninsured. Neighborhood deprivation used the 2023 Area Deprivation Index (ADI-NatRank) from the Neighborhood Atlas [20]. Distance to center was calculated from ZIP centroid coordinates using the haversine method [21].

### Surgical technique

Operations were performed by pituitary neurosurgeons using an endoscopic endonasal TSS approach with selective adenoma resection guided by preoperative MRI, anatomic landmarks, and neuronavigation when available. With radiographic or intraoperative cavernous sinus invasion (CSI), selective resection of the medial wall was considered per consensus recommendations [22, 23].

### Covariates

Preoperative variables included age, sex, BMI, hypertension, diabetes mellitus, obstructive sleep apnea, obesity (BMI ≥ 30 kg/m²), neurological symptoms, and Karnofsky Performance Status (KPS). Preoperative MRI variables included tumor volume (ellipsoid formula), macroadenoma (≥ 1 cm), Knosp grade (0–4), CSI (Knosp 3–4), optic chiasm compression, contrast enhancement, and morphology (solid, cystic, mixed) [24]. Endocrinologic presentation used preoperative serum cortisol (SC), plasma ACTH, LNSC, and UFC [18, 25].

### Outcomes

Operative outcomes were subtotal resection (STR) and cerebrospinal fluid (CSF) leak requiring intervention within 30 days [9]. Perioperative endocrine outcomes were acute postoperative AVP-D, defined as new hypotonic polyuria and/or hypernatremia requiring desmopressin during hospitalization or within 30 days and classified as transient if it resolved during follow-up or permanent if desmopressin was still required at last follow-up, panhypopituitarism, defined as new deficiency of at least three of the anterior pituitary axes (corticotroph, thyrotroph, gonadotroph, somatotroph, and lactotroph), each based on postoperative dynamic or basal hormone testing below institutional reference thresholds or a documented requirement for hormone replacement, evaluated at the most recent endocrine assessment within the 12-month follow-up window [17], and day 1 postoperative morning SC measured prior to glucocorticoid administration [26].

Postoperative normocortisolism was SC < 5 µg/dL [27, 28]. Durable biochemical remission (BR) was sustained suppression at 12 months without steroidogenesis inhibitor use or repeat surgery, confirmed by SC < 2 µg/dL and normalization of UFC, LNSC, or DST on longitudinal testing per Pituitary Society guidance [18, 29–31]. First remission was stratified as early (normocortisolism within 72 h or BR within 30 days) or late (≥ 6 months postoperatively, consistent with delayed HPA axis recovery [32]); consensus criteria for early versus late remission are non-standardized.

Survival outcomes were radiographic disease and reintervention. Radiographic DFS was the absence of residual or recurrent tumor on surveillance MRI, with STR or recurrence after gross total resection (GTR) as disqualifying events. MRI surveillance occurred at 1, 3, 6, and 12 months postoperatively [23, 33]; patients without later imaging were censored unless intervention occurred. Reintervention included repeat pituitary surgery or radiotherapy during 12-month follow-up. Reintervention-free survival (RFS) was time from primary TSS to reintervention or last clinical follow-up, with administrative censoring at 12 months; reintervention events occurring beyond 12 months were administratively recoded to day 365 to align with the 12-month analytic horizon.

### Statistical analysis

The primary analysis was descriptive. Omnibus comparisons used Kruskal-Wallis tests for continuous variables and chi-square or Fisher’s exact tests for categorical variables; pairwise comparisons used Mann-Whitney U or Fisher’s exact tests. Continuous variables are reported as median (IQR) and categorical as n (%). Unadjusted effect estimates used logistic regression for binary outcomes, Cox proportional hazards for time-to-event, and ordinary least squares for continuous outcomes; estimates are odds ratios (OR), hazard ratios (HR), or regression coefficients with 95% CI and two-sided *p* < 0.05. The primary analysis remained descriptive and unadjusted because of limited subgroup size; IPTW was performed solely as an exploratory sensitivity analysis. IPTW used a multinomial logistic propensity model including age, sex, BMI, insurance, distance to center, ADI national rank, comorbidity count, and KPS; stabilized weights were truncated at the 1st-99th percentiles. Weighted logistic, linear, and Cox models with robust standard errors were used for outcomes, and absolute standardized mean differences (aSMD) assessed balance. Given the small Black subgroup (*n* = 16) and residual post-weighting imbalance, IPTW estimates for Black patients are exploratory. Analyses used Python 3.12.

## Results

### Overall cohort characteristics

A total of 104 patients underwent primary TSS for CD: 44 WnH (42%), 44 Hispanic (42%), and 16 Black (15%). Median age was 48 years (IQR 36–60), 79% female, median BMI 30.7 kg/m² (Table 1). Hypertension (61%) and obesity (54%) were the most common comorbidities. Median preoperative KPS was 90 (IQR 80–90); 71% had private insurance, 23% Medicare/Medicaid, 5.8% uninsured. Patients lived a median of 24.3 miles (IQR 13.1–55.8) from the surgical center, with significant group differences (*p* = 0.003). ADI national rank and insurance distribution did not differ across groups (*p* = 0.459, *p* > 0.1). Median tumor volume was 0.2 cm³ (IQR 0.0-1.1); 27% were macroadenomas, 7.7% occult, 27% demonstrated CSI, and 21% chiasmal compression. Tumors were predominantly solid (66%), 21% mixed, 13% cystic. Preoperative biochemistry showed median SC 14.8 µg/dL (IQR 9.2–24.4), ACTH 43.5 pg/mL (IQR 20.8–83.6), and UFC 121.6 µg/24 h (IQR 65.3-284.5). LNSC was obtained in 44%, IPSS in 38%, and 24-hour UFC in 34%; availability did not differ across groups (all *p* > 0.1; Supplemental Table 1).

**Table 1:** Baseline characteristics of the cohort by race and ethnicity

Characteristics	Overall (*N* = 104)	WnH (*n* = 44)	Hispanic (*n* = 44)	Black (*n* = 16)	*p*-value
**Comorbidities and function**					
Age at surgery (years)	48.0 (36.0–60.0)	59.0 (38.0–64.0)	45.0 (37.0–52.0)	36.0 (26.0–46.0)	0.003**
Male sex	22 (21%)	15 (34%)	5 (11%)	2 (13%)	0.022*
BMI (kg/m²)	30.7 (25.9–36.9)	28.5 (25.1–32.6)	31.8 (25.8–39.9)	33.3 (26.5–39.9)	0.033*
Comorbidity count	2.0 (1.0–3.0)	1.0 (1.0–2.0)	2.0 (1.0–3.0)	2.0 (1.0–3.0)	0.183
Hypertension	63 (61%)	26 (59.1%)	24 (55%)	13 (81%)	0.167
Diabetes mellitus	47 (45%)	19 (43%)	18 (41%)	10 (63%)	0.311
Obstructive sleep apnea	12 (12%)	7 (16%)	4 (9.1%)	1 (6.2%)	0.468
Obesity	56 (54%)	18 (41%)	27 (61%)	11 (69%)	0.067†
Preoperative KPS	90.0 (80.0–90.0)	90.0 (80.0–90.0)	90.0 (80.0–90.0)	90.0 (90.0–90.0)	0.504
**Access to care**					
Private insurance	74 (71%)	28 (64%)	35 (80%)	11 (69%)	0.251
Public insurance	24 (23%)	11 (25%)	8 (18%)	5 (31%)	0.525
Uninsured	6 (5.8%)	5 (11%)	1 (2.3%)	0 (0.0%)	0.105
ADI national rank	34.8 (22.5–44.0)	34.0 (16.0–46.0)	34.5 (24.0–41.0)	38.0 (27.0–53.0)	0.459
Distance to center (miles)	24.3 (13.1–55.8)	35.3 (16.6–78.2)	15.7 (10.3–31.1)	29.6 (13.1-107.2)	0.003**
Presenting symptoms					
Headache	12 (12%)	6 (14%)	4 (9.1%)	2 (13%)	0.794
Visual symptoms	24 (23%)	13 (30%)	8 (18%)	3 (19%)	0.407
Cognitive disturbance	11 (11%)	6 (14%)	5 (11%)	0 (0.0%)	0.308
**Endocrinologic presentation**					
Serum cortisol (µg/dL)	14.8 (9.2–24.4)	15.1 (12.0-23.1)	13.9 (7.7–23.7)	18.6 (9.4–39.7)	0.132
Plasma ACTH (pg/mL)	43.5 (20.8–83.6)	40.8 (19.0–84.0)	35.0 (20.6–84.9)	53.3 (43.0-81.1)	0.596
LNSC (µg/dL)	0.2 (0.1–0.4)	0.3 (0.2-1.0)	0.2 (0.1–0.2)	0.3 (0.2–0.4)	0.074†
24-hour UFC (µg/24 h)	121.7 (65.3-284.5)	119.4 (65.8–340.0)	105.2 (60.0-200.0)	115.0 (75.2–582.0)	0.641
**Radiographic presentation**					
Tumor volume (cm³)	0.2 (0.0-1.1)	0.2 (0.0-1.3)	0.2 (0.0-0.6)	0.2 (0.1–0.7)	0.906
Macroadenoma	28 (27%)	14 (32%)	10 (23%)	4 (25%)	0.619
Occult tumor	8 (7.7%)	5 (11%)	3 (6.8%)	0 (0.0%)	0.330
CSI	28 (27%)	13 (30%)	12 (27%)	3 (19%)	0.705
Enhancement	62 (60%)	24 (55%)	26 (59%)	12 (75%)	0.359
Chiasmal compression	22 (21%)	10 (23%)	9 (21%)	3 (19%)	0.935
Tumor morphology	-	-	-	-	0.040*
*Solid*	69 (66%)	27 (61%)	27 (61%)	15 (94%)	-
*Cystic*	13 (13%)	7 (16%)	6 (14%)	0 (0.0%)	-
*Mixed*	22 (21%)	10 (23%)	11 (25%)	1 (6.2%)	-

Demographic, socioeconomic, comorbidity, presenting symptom, endocrinologic, and radiographic characteristics for the full cohort and stratified by race and ethnicity. Continuous variables are presented as median (interquartile range); categorical variables as n (%). P-values: Kruskal-Wallis test for continuous variables; chi-square or Fisher's exact test for categorical variables

^1^ *p < 0.05; **p < 0.01; ***p < 0.001; † p < 0.10. wnh = white non-hispanic; adi = area deprivation index; bmi = body mass index; kps = karnofsky performance status; lnsc = late-night salivary cortisol; ufc = urinary free cortisol; csi = cavernous sinus invasion

### Sensitivity IPTW Analysis

The multinomial propensity score model demonstrated adequate fit (McFadden pseudo-R²=0.20, Nagelkerke R²=0.39, AUC = 0.78); stabilized weights were acceptable without major truncation (Supplemental Appendix Fig. 1, Supplemental Table 2).

### Hispanic patients present with a more favorable baseline phenotype

Hispanic patients presented with a more favorable baseline profile (Table 1). They lived closest to the surgical center (median 15.7 miles vs. 35.3 WnH and 29.6 Black; *p* = 0.003), with numerically milder biochemistry (lowest median SC, LNSC, and UFC) and trends toward better insurance access (80% private insurance; 2.3% uninsured) versus WnH, none reaching significance. Age, BMI, comorbidities, and tumor morphology did not significantly differ from WnH. After IPTW, WnH and Hispanic patients were broadly comparable, with most variables achieving aSMD < 0.20 (Supplemental Appendix Fig. 1, Supplemental Table 3); modest residual imbalance persisted in hypertension (aSMD 0.31), ADI national rank (aSMD 0.25), and distance to center (aSMD 0.20), and biochemical markers remained imbalanced in the direction of milder Hispanic disease (LNSC aSMD 0.87, plasma ACTH aSMD 0.33; both higher in WnH), with cystic morphology showing modest residual imbalance (aSMD 0.23).

### Black patients present with a more severe baseline phenotype

Black patients were youngest (median 36 years; *p* = 0.003), with highest BMI (33.3 kg/m²; *p* = 0.033) and lived numerically furthest from the surgical center (29.6 miles). Tumor morphology was the only radiographic variable to differ across groups (*p* = 0.040): Black patients had near-uniformly solid tumors (94%, with 0% cystic, 6.2% mixed) versus 61% solid in WnH and Hispanic patients. Biochemical severity was numerically highest in Black patients, with a borderline global difference for LNSC (*p* = 0.074; Table 1). After IPTW, balance between WnH and Black patients was substantially more limited, with persistent imbalance across comorbidities, public insurance, and cognitive disturbance (aSMDs 0.43–0.60), radiographic features (tumor enhancement aSMD 0.65, chiasmal compression aSMD 0.44, fewer occult tumors aSMD 0.47), and tumor morphology (solid aSMD 0.84). Biochemical severity also remained substantially imbalanced (SC aSMD 0.79, ACTH 0.80, UFC 0.55), indicating that baseline disease severity could not be fully balanced by weighting and adjusted estimates for Black patients remain susceptible to residual confounding (Supplemental Appendix Fig. 1; Supplemental Table 3). Given *n* = 16 and residual post-weighting imbalance, IPTW estimates for Black versus WnH comparisons are exploratory.

### Overall cohort outcomes and complications

GTR was achieved in 89% (*n* = 92). By last follow-up, durable BR was achieved in 83% of the cohort (Table 2). Throughout follow-up, persistent radiographic disease in 22% overall. Reintervention occurred in 8.7% of the cohort (*n* = 9). CSF leak was comparable across groups (WnH 43%, Hispanic 57%, Black 56%; all pairwise *p* > 0.2). Panhypopituitarism was uncommon (6.7%, *n* = 7). Postoperative AVP-D occurred in 41% overall (*n* = 43). No mortality was observed.

**Table 2:** Perioperative and longitudinal outcomes by race and ethnicity, before and after IPTW adjustment

	WnH	Hispanic	Black	OR/β (95% CI), *p*	
				Unadjusted	After IPTW
**A. Perioperative Outcome**					
**Hispanic vs. WnH**					
CSF leak	43% (19)	57% (25)	-	1.73 (0.74–4.02), *p* = 0.202	1.86 (0.78–4.42), *p* = 0.161
AVP-D	55% (24)	25% (11)	-	0.28 (0.11–0.69), *p* = 0.005**	0.18 (0.07–0.46), *p* < 0.001***
Panhypopituitarism	2.3% (1)	6.8% (3)	-	3.15 (0.31–31.49), *p* = 0.329	2.67 (0.33–21.57), *p* = 0.356
* Length of stay (days)*	3.5 (3.0–5.0)	4.0 (3.0–5.0)	-	-1.07 (-3.88-1.25), *p* = 0.363	-0.47 (-2.28-1.34), *p* = 0.608
**Black vs. WnH**					
CSF leak	43% (19)	-	56% (9)	1.69 (0.53–5.36), *p* = 0.372	2.23 (0.62-8.00), *p* = 0.218
AVP-D	55% (24)	-	50% (8)	0.83 (0.27–2.62), *p* = 0.755	0.43 (0.12–1.53), *p* = 0.194
Panhypopituitarism	2.3% (1)	-	19% (3)	9.92 (0.95-103.72), *p* = 0.055†	6.58 (0.68–64.02), *p* = 0.104
* Length of stay (days)*	3.5 (3.0–5.0)	-	4.0 (3.0–6.0)	-0.19 (-3.37-2.98), *p* = 0.904	2.28 (-0.36-4.92), *p* = 0.090†
**B. Longitudinal Outcome**					
**Hispanic vs. WnH**					
**Biochemical remission**					
* Day 1 postop SC µg/dL*	7.0 (3.0-22.8)	6.8 (1.9–19.2)	-	-1.94 (-7.81-3.92), *p* = 0.512	-0.23 (-6.91-6.45), *p* = 0.945
Durable BR	79%	81%	-	1.16 (0.40–3.35), *p* = 0.787	0.72 (0.27–1.96), *p* = 0.523
* Early*	52%	43%	-	0.69 (0.30–1.61), *p* = 0.394	0.73 (0.31–1.73), *p* = 0.469
* Late*	23%	40%	-	2.16 (0.85–5.50), *p* = 0.107	1.72 (0.65–4.54), *p* = 0.271
Durable BR time-to-event	7.0 (3.0-22.8)	6.8 (1.9–19.2)	-	1.03 (0.66–1.61), *p* = 0.905	0.91 (0.50–1.66), *p* = 0.769
**Radiographic disease**					
Subtotal resection	9.1% (4)	11% (5)	-	1.28 (0.32–5.13), *p* = 0.725	2.66 (0.66–10.62), *p* = 0.167
Radiographic persistence	21%	18%		0.86 (0.30–2.49), *p* = 0.787	1.91 (0.70–5.24), *p* = 0.209
DFS time-to-event	-	-		0.87 (0.35–2.19), *p* = 0.773	1.70 (0.66–4.35), *p* = 0.271
Need for reintervention					
Reintervention	4.5%	6.8%		1.54 (0.24–9.68), *p* = 0.647	6.89 (0.95–50.26), *p* = 0.057†
RFS time-to-event	-	-		1.31 (0.28–6.23), *p* = 0.735	4.20 (1.10-16.08), *p* = 0.036*
Black vs. WnH					
**Biochemical remission**					
* Day 1 postop SC µg/dL*	7.0 (3.0-22.8)	-	15.3 (6.9–22.3)	5.05 (-3.01-13.10), *p* = 0.217	16.06 (6.05–26.07), *p* = 0.002*
Durable BR	79%	-	88%	1.86 (0.37–9.43), *p* = 0.451	5.19 (0.42–63.7), *p* = 0.190
* Early*	52%	-	44%	0.70 (0.24–2.05), *p* = 0.516	0.44 (0.13–1.48), *p* = 0.190
* Late*	23%	-	44%	2.57 (0.74–8.97), *p* = 0.140	5.76 (1.65–20.2), *p* = 0.006**
Durable BR time-to-event				1.88 (0.68–5.18), *p* = 0.221	2.83 (0.80–9.97), *P* = 0.105
**Radiographic disease**					
Subtotal resection	9.1% (4)	-	19% (3)	2.31 (0.46–11.69), *p* = 0.312	1.08 (0.11–10.87), *p* = 0.945
Radiographic persistence	21%	-	38%	2.33 (0.67–8.12), *p* = 0.184	3.82 (1.00-14.53), *p* = 0.049*
DFS time-to-event	-	-	-	1.88 (0.68–5.18), *p* = 0.221	2.83 (0.80–9.97), *P* = 0.105
**Need for reintervention**					
Reintervention	4.5%	-	25%	7.00 (1.14–42.97), *p* = 0.036*	4.95 (0.43–57.55), *p* = 0.201
RFS time-to-event	-	-	-	4.68 (1.02–21.54), *p* = 0.048*	2.88 (0.74–11.13), *p* = 0.125

(A) Perioperative complications and outcomes including subtotal resection, cerebrospinal fluid leak, postoperative arginine vasopressin deficiency, panhypopituitarism, and length of stay, with pairwise comparisons of Hispanic versus WnH and Black versus WnH patients before and after IPTW adjustment. (B) Day-1 postoperative serum cortisol, durable biochemical remission (early and late phenotypes), subtotal resection, radiographic persistence, disease-free survival, reintervention, and reintervention-free survival, with pairwise comparisons of Hispanic versus WnH and Black versus WnH patients before and after IPTW adjustment

^1^ Odds ratios derived from logistic regression for binary outcomes; coefficient β from ordinary least squares regression for the continuous outcome; length of stay. IPTW estimates use a multinomial propensity model with stabilized weights truncated at the 1^st^-99th percentiles; weighted models use robust standard errors*p < 0.05; **p < 0.01; ***p < 0.001; † p < 0.10. or = odds ratio; hr = hazard ratio; ci = confidence interval; iptw = inverse probability of treatment weighting; wnh = white non-hispanic; csf = cerebrospinal fluid; avp-d = arginine vasopressin deficiency; br = biochemical remission; sc = serum cortisol; dfs = disease-free survival; rfs = reintervention-free survival

### Durable remission rates were comparable across groups

Durable BR was achieved in 83% of the cohort (Table 2), with broadly similar rates across groups (WnH 79%, Hispanic 81%, Black 88%; pairwise *p* = 0.787 Hispanic vs. WnH; *p* = 0.451 Black vs. WnH).

### Black patients showed higher postoperative biochemical severity and delayed remission

**Fig. 1:**
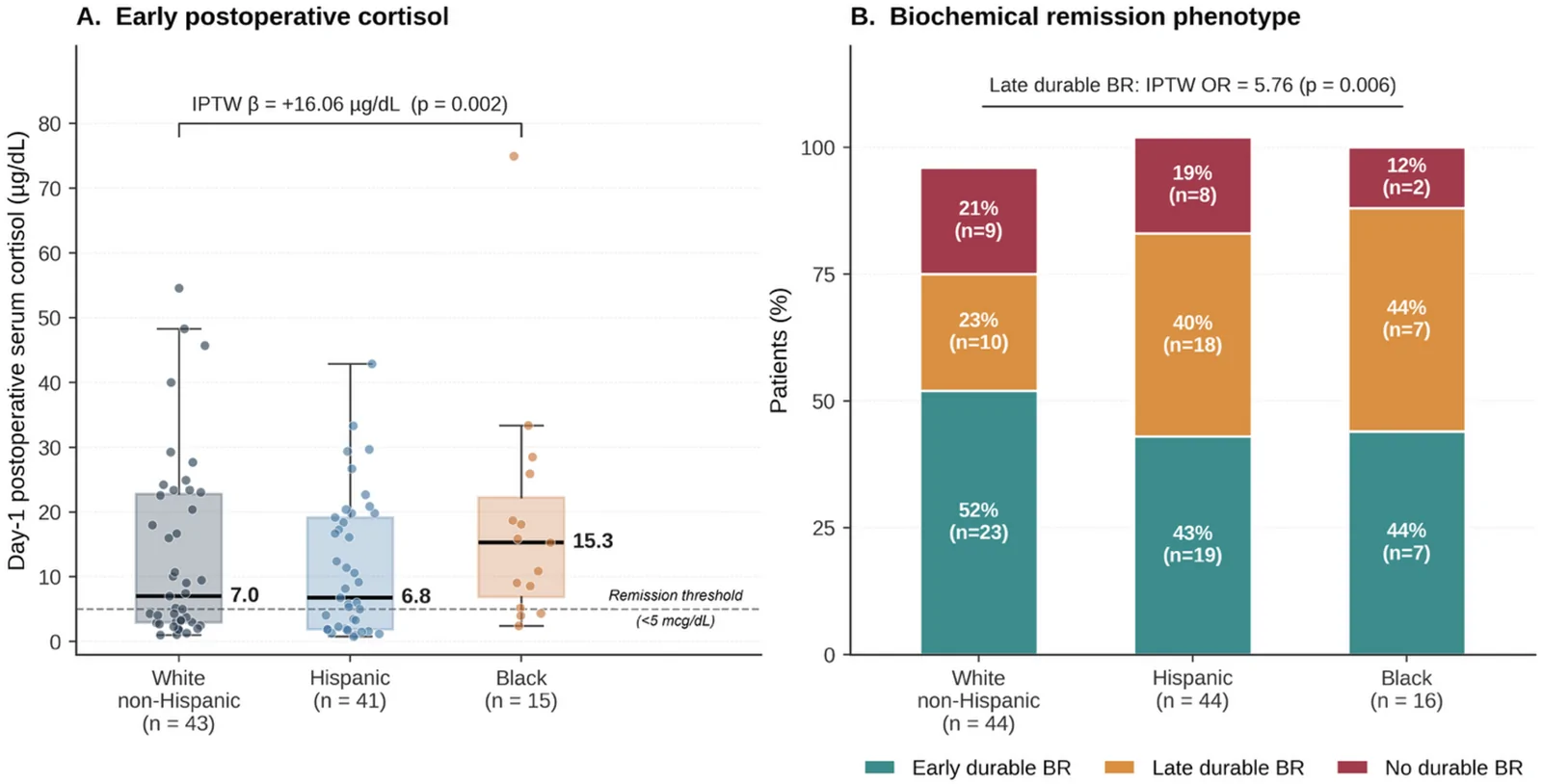
Postoperative cortisol trajectory and biochemical remission phenotype by race and ethnicity. (**A**) Day-1 postoperative serum cortisol by race and ethnicity. Boxes show median and interquartile range; whiskers extend to 1.5× IQR. Individual patient values overlaid as jittered points. The dashed reference line at 5 µg/dL marks the consensus remission threshold. The bracket reports the IPTW-adjusted between-group regression coefficient for Black versus WnH patients. (**B**) Distribution of biochemical remission (BR) phenotype by race and ethnicity. Patients are categorized as: early durable BR (nadir SC < 5 µg/dL within 72 h or remission within 30 days, with sustained durable BR); late durable BR (durable BR first documented ≥ 6 months postoperatively); or no durable BR. The bracket reports the IPTW-adjusted odds ratio for late durable BR in Black versus WnH patients. Effect estimates derived from IPTW-adjusted models with stabilized weights truncated at the 1st-99th percentiles. a iptw = inverse probability of treatment weighting; wnh = white non-hispanic; br = biochemical remission

Despite comparable durable BR rates, biochemical trajectories differed (Fig. 1A, B). Day-1 postoperative SC was numerically higher in Black than WnH patients unadjusted (median 15.3 vs. 7.0 µg/dL; β + 5.05 µg/dL, 95% CI -3.01 to 13.10; *p* = 0.217). Among patients reaching durable BR, late rather than early remission was numerically more common in Black than WnH patients (44% vs. 23%; unadjusted OR 2.57, 95% CI 0.74–8.97; *p* = 0.140). Day-1 SC and BR timing did not differ significantly between Hispanic and WnH patients. On IPTW sensitivity, Black versus WnH differences in day-1 SC (β + 16.06 µg/dL, 95% CI 6.05–26.07; *p* = 0.002) and late durable remission (IPTW OR 5.76, 95% CI 1.65–20.20; *p* = 0.006) reached significance but are limited by small sample size and residual imbalance after weighting.

### Hispanic patients had lower postoperative arginine vasopressin deficiency

Postoperative AVP-D was significantly less frequent in Hispanic than WnH patients (25% vs. 55%; OR 0.28, 95% CI 0.11–0.69; *p* = 0.005). AVP-D rates were similar between Black and WnH patients (50% vs. 55%; OR 0.83, 95% CI 0.27–2.62; *p* = 0.755). On IPTW sensitivity, the Hispanic versus WnH AVP-D difference persisted (IPTW OR 0.18, 95% CI 0.07–0.46; *p* < 0.001). Of the 43 patients with postoperative AVP-D, most were transient; permanent AVP-D requiring ongoing desmopressin at last follow-up occurred in 5 patients (WnH 3 [6.8%], Hispanic 1 [2.3%], Black 1 [6.2%]).

### Black patients had higher radiographic persistence of disease

**Fig. 2:**
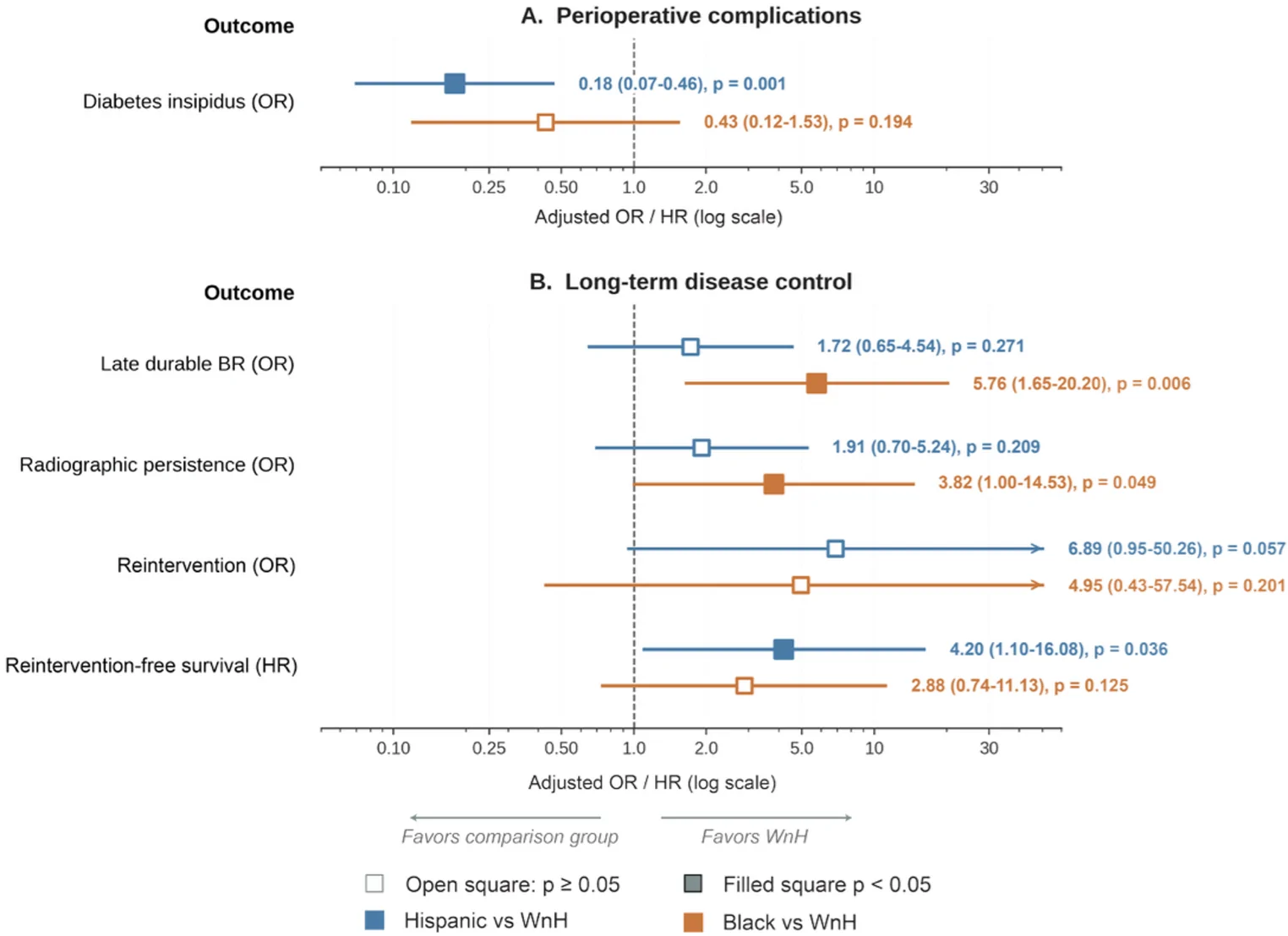
IPTW-adjusted effect estimates for primary perioperative and disease-control outcomes by race and ethnicity. Forest plot of IPTW-adjusted odds ratios (binary outcomes) and hazard ratios (time-to-event outcomes) for (**A**) Hispanic versus White non-Hispanic patients and (**B**) Black versus White non-Hispanic patients. Squares represent point estimates; horizontal bars represent 95% confidence intervals. Filled squares indicate *p* < 0.05. Arrowheads at the right margin denote upper confidence limits exceeding the plotted scale. All estimates derived from IPTW-weighted regression models. Logistic regression was used for binary outcomes (subtotal resection, CSF leak, arginine vasopressin deficiency, panhypopituitarism, durable biochemical remission, late durable biochemical remission, radiographic persistence, reintervention). Cox proportional hazards regression was used for time-to-event outcomes (reintervention-free survival, disease-free survival). Stabilized weights were truncated at the 1st -99th percentiles; weighted models use robust standard errors. ^1^ br = biochemical remission; ci = confidence interval; csf = cerebrospinal fluid; hr = hazard ratio; iptw = inverse probability of treatment weighting; or = odds ratio; wnh = white non-hispanic

Persistent radiographic disease was higher in Black patients (38%) than WnH (21%) or Hispanic (18%); the unadjusted Black versus WnH comparison was not significant (OR 2.33, 95% CI 0.67–8.12; *p* = 0.184). On IPTW sensitivity, the Black versus WnH difference in radiographic persistence reached significance (IPTW OR 3.82, 95% CI 1.00-14.53; *p* = 0.049), with a non-significant Cox trend (IPTW HR 2.83, 95% CI 0.80–9.97; *p* = 0.105; Fig. 2). Adjusted estimates for the Black subgroup are exploratory.

### Shorter reintervention-free survival signal in both non-white subgroups

**Fig. 3:**
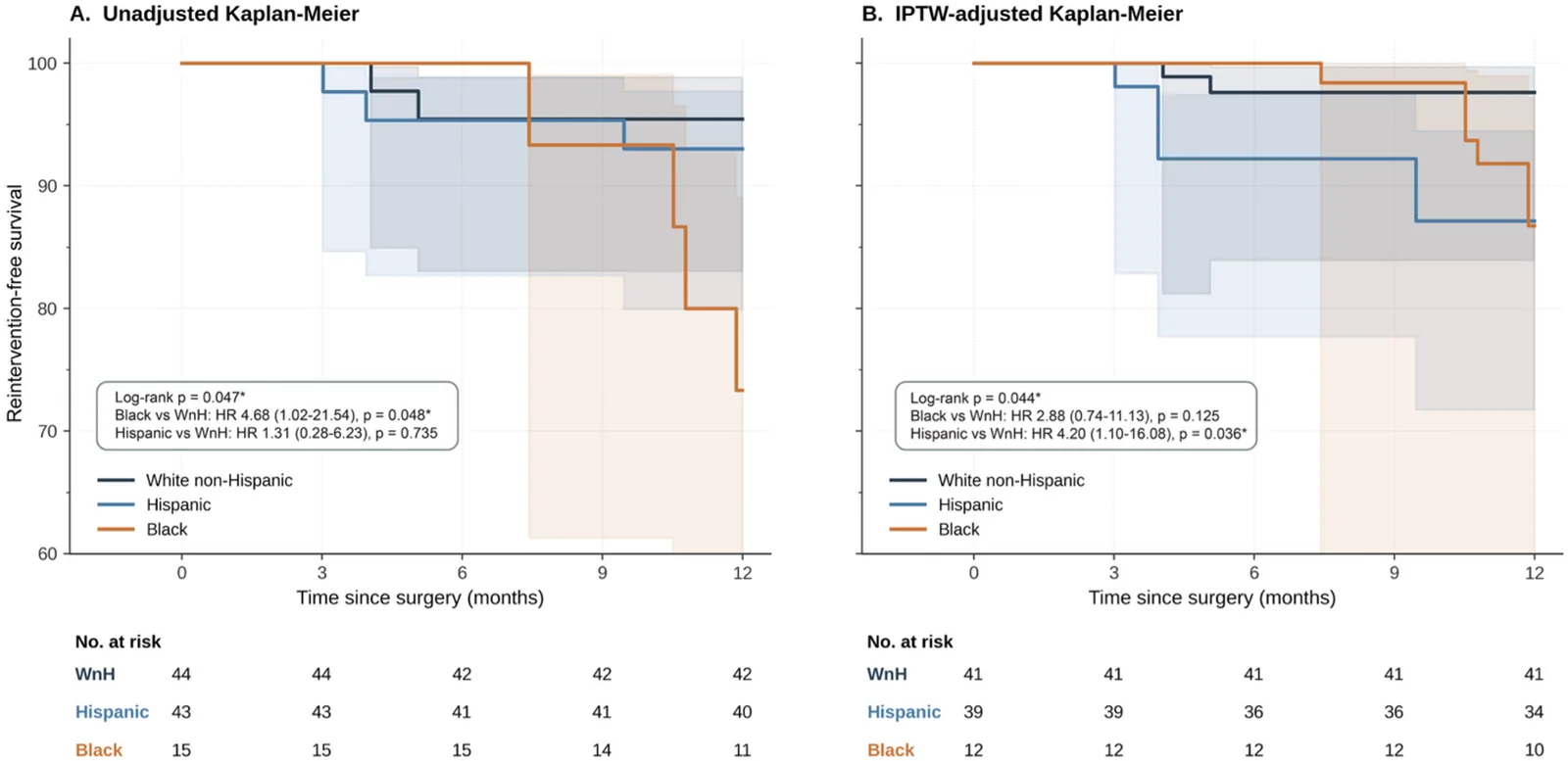
Reintervention-free survival after primary transsphenoidal surgery for Cushing’s disease, before and after IPTW adjustment. (**A**) Unadjusted Kaplan-Meier estimates of reintervention-free survival from the date of primary transsphenoidal surgery. (**B**) IPTW-adjusted Kaplan-Meier estimates using stabilized inverse-probability-of-treatment weights from the multinomial propensity model. Risk tables below each panel show the unweighted (**A**) and effective weighted (**B**) number of patients at risk at each annual interval. Hazard ratios and log-rank p-values are shown in each panel. Reintervention defined as repeat pituitary surgery or radiotherapy during follow-up, per the Methods. Patients without a reintervention event are censored at the date of last clinical follow-up. Curves are plotted on a 12-month horizon to align with the primary follow-up window for reintervention; events occurring after 12 months were administratively censored as events at day 365. The propensity model for IPTW included age, sex, BMI, insurance type, distance to the surgical center, ADI national rank, comorbidity count, and Karnofsky Performance Status; weights were stabilized and truncated at the 1st -99th percentiles. ^a^ ci = confidence interval; hr = hazard ratio; iptw = inverse probability of treatment weighting; wnh = white non-hispanic

Crude reintervention rates differed across groups: 4.5% WnH, 6.8% Hispanic, 25% Black (global *p* = 0.038). Black patients had significantly higher unadjusted odds of reintervention versus WnH (OR 7.00, 95% CI 1.14–42.97; *p* = 0.036). Unadjusted RFS also differed (log-rank *p* = 0.047), with Black patients showing significantly shorter RFS than WnH (HR 4.68, 95% CI 1.02–21.54; *p* = 0.048; Fig. 3A). On IPTW sensitivity, both non-White groups showed shorter RFS than WnH: the Hispanic versus WnH comparison reached significance (IPTW HR 4.20, 95% CI 1.10-16.08; *p* = 0.036) and the adjusted global log-rank remained significant (*p* = 0.044), with curves demonstrating separation between WnH and non-White patients (Fig. 3B). IPTW estimates for the Black subgroup are exploratory.

## Discussion

Our institution serves a racially and ethnically diverse area, including approximately 13% Black, 72% Hispanic, and 23% White residents, with 12% WnH, providing regional context for assessing differences within a shared tertiary referral network [34]. To our knowledge, this is the first study evaluating whether race and ethnicity are associated with baseline phenotype, perioperative endocrine morbidity, and long-term disease control after TSS for CD [35]. Organizing these findings along the surgical care pathway, differences emerged before surgery in age, BMI, tumor morphology, and access characteristics; early after surgery in arginine vasopressin deficiency and day-1 cortisol; and over long-term follow-up, where durable biochemical remission converged across groups while surveillance and reintervention trajectories diverged. However, Black subgroup analyses were limited by small sample size and residual imbalance after weighting, and adjusted estimates in this group are exploratory and hypothesis-generating rather than evidence of independent association. Descriptive findings, which do not depend on the propensity model, were broadly consistent with the IPTW sensitivity analysis.

Hispanic patients presented with the most favorable baseline profile, living closer to the surgical center and demonstrating the highest rate of private insurance, with lower SC and ACTH and fewer invasive radiographic features than WnH patients. Postoperative AVP-D was less frequent in Hispanic than WnH patients, a finding that persisted on IPTW sensitivity and is consistent with prior reports [36], though contrary to others [37]; tumor volume and CSI did not differ materially between groups, limiting inferences based on structural severity. Despite favorable early and access features, Hispanic patients ultimately showed a signal toward worse longer-term disease control, with shorter RFS on IPTW sensitivity paralleling national database studies of minority populations [3]. This reintervention-free survival signal was not evident in unadjusted analysis (HR 1.31; *p* = 0.735) and emerged only after weighting, and should therefore be regarded as exploratory. This pattern suggests early access advantages may mask less favorable longitudinal trajectories.

Black patients differed markedly from WnH patients at baseline by younger age and higher BMI, with numerically elevated SC and ACTH and near-uniformly solid tumor morphology, may reflect differences in access or referral pathways and potential barriers including public insurance. National database and pediatric literature have similarly reported lower surgical utilization and greater CD severity at presentation among Black and Hispanic patients [38, 39]. Crude reintervention rates were highest in Black patients (25%), and unadjusted RFS was significantly shorter than in WnH patients. On IPTW sensitivity, Black patients also demonstrated higher day-1 postoperative SC, were more likely to achieve late rather than early durable remission, and had higher odds of persistent radiographic disease, directionally concordant with delayed endocrinologic resolution and consistent with persistent early hypercortisolism [40, 41]. Tumor morphology showed a large and persistent disparity, with solid morphology more common in Black patients and unchanged after IPTW, a feature previously associated with poorer remission [42–45] and not explained by measured covariates.

Several mechanisms may underlie these race- and ethnicity-associated differences, none of which can be resolved in the present cohort. Biologically, the near-uniformly solid tumor morphology and higher postoperative cortisol observed in Black patients could reflect more invasive corticotroph phenotypes or later presentation with greater disease burden, although tumor volume did not differ significantly across groups. Structurally, differences in referral timing, geographic access, insurance type, and neighborhood deprivation may delay diagnosis and specialty endocrine follow-up, contributing to later remission and higher radiographic persistence. Additional unmeasured contributors, including language concordance, health literacy, and adherence to postoperative biochemical and imaging surveillance, may further shape divergent longitudinal trajectories despite comparable ultimate remission. Evolving cortisol and ACTH assay sensitivity across the study period may also have influenced absolute biochemical values and their interpretation. Disentangling biological from access-related contributions will require larger, prospectively harmonized multicenter cohorts with granular socioeconomic and operative data.

Clinically, postoperative endocrine surveillance may need to be tailored by risk profile, including race- and ethnicity-associated presentation patterns. For Hispanic patients, lower AVP-D does not necessarily translate to lower long-term disease risk, supporting careful biochemical surveillance regardless of early endocrine morbidity. For Black patients, higher day-1 SC, delayed remission, and higher radiographic persistence suggest standardized biochemical monitoring, earlier endocrine review and surveillance imaging, and lower thresholds for adjuvant therapy when early SC remains elevated. Persistence of tumor morphology differences raises the possibility that intraoperative tissue characteristics contribute to differential outcomes and warrants evaluation of medial wall or dural involvement in future studies. The RAPID multicenter registry did not detect race as a univariate predictor of recurrence and did not include race in multivariable modeling [31, 35]. Future multicenter studies should incorporate operative variables, demographic covariates, and prospective harmonization of biochemical testing and remission definitions [46].

This study evaluated a racially and ethnically diverse cohort at a single high-volume center, standardizing operative and perioperative pathways relative to administrative datasets. Pathologic confirmation of corticotroph adenomas strengthened diagnostic specificity [19]. We incorporated neighborhood deprivation and geographic distance, dimensions often absent from pituitary surgical outcomes literature [47].

Several limitations warrant consideration. First, this retrospective single-center study remains susceptible to residual confounding from unmeasured variables, including referral patterns, language concordance, health literacy, follow-up adherence, and surgeon-specific variation. Second, the Black subgroup (*n* = 16) was small, with low event counts and residual post-weighting imbalance in clinical, biochemical, and radiographic covariates; IPTW estimates in this subgroup are likely underpowered and exploratory rather than evidence of independent association. Type II error cannot be excluded for large but nonsignificant effect sizes, and these comparisons require validation in larger multicenter cohorts [48]. Race and ethnicity were self-reported and categorical, and mixed-race patients were not specifically assessed. Third, this cohort spans 2013–2024, over which institutional cortisol and ACTH assays evolved toward higher analytical sensitivity, particularly after 2019; an era effect on absolute biochemical values cannot be fully excluded [49]. Fourth, tumor morphology variables were derived from clinical MRI interpretation and are subject to measurement variability, and intraoperative confirmation of dural or CSI was not uniformly recorded. Fifth, retreatment decisions reflect clinical judgment and evolving endocrine management, and management-driven variation cannot be excluded. Sixth, although postoperative arginine vasopressin deficiency was characterized as transient or permanent, the anterior pituitary deficits contributing to panhypopituitarism were ascertained at the last available endocrine assessment within 12 months and were not further classified as transient or permanent, which may misclassify long-term anterior pituitary morbidity. Seventh, several associations reached significance only after IPTW while remaining nonsignificant in unadjusted analysis, and given the small subgroups and residual imbalance these weighted estimates may be unstable and warrant cautious, hypothesis-generating interpretation. Finally, these findings are hypothesis-generating associations requiring confirmation in larger prospective multicenter studies rather than evidence of intrinsic racial differences.

## Conclusion

In this single-center cohort undergoing TSS for CD, baseline presentation differed substantially across racial and ethnic groups, whereas durable biochemical remission converged. Differences emerged primarily along the care pathway, in early postoperative recovery and long-term surveillance trajectory, rather than in ultimate endocrine control. Hispanic patients presented with a more favorable baseline profile, including lower postoperative AVP-D, but showed a signal toward worse longer-term disease control on exploratory adjusted analysis. Black patients presented with a more severe baseline phenotype and showed higher radiographic persistence and crude reintervention rates, with exploratory adjusted analyses suggesting higher postoperative biochemical severity and delayed remission. Black subgroup analyses were limited by small sample size and should be considered hypothesis-generating. Structural and access factors may obscure true differences in postoperative trajectory; these findings support tailored postoperative surveillance and warrant validation in larger multicenter studies.

## Supplementary Information

Below is the link to the electronic supplementary material.


Supplementary Material 1


## Data Availability

No datasets were generated or analysed during the current study.
