# Supplementary material for: Racial and ethnic differences in presentation and postoperative recovery after transsphenoidal surgery for cushing disease
Source: J Neurooncol. 2026 Aug 6;179(1):31. doi: 10.1007/s11060-026-05741-x (PMC13447533; doi:10.1007/s11060-026-05741-x)
Supplement: Supplementary file 1 [file 11060_2026_5741_MOESM1_ESM.docx]

**SUPPLEMENTARY DATA**

**Supplemental Appendix Table 1. Availability of preoperative biochemical testing by race and ethnicity**

Proportion of patients with each preoperative biochemical assay obtained and results of sensitivity analysis, by race and ethnicity. *Data shown as % (n). P-values: chi-square test. ^1^ acth = adrenocorticotropic hormone; lmsc = late-night salivary cortisol; ufc = urinary free cortisol.*

| **Biochemical assay** | **Cohort** | **WnH** | **Hispanic** | **Black** | **𝜒**2 | ***p*-value** |
| --- | --- | --- | --- | --- | --- | --- |
| Serum cortisol, µg/dL | 78% (81) | 86% (38) | 71% (31) | 75% (12) | 3.32 | 0.190 |
| Plasma ACTH, pg/mL | 80% (83) | 86% (38) | 77% (34) | 69% (11) | 2.56 | 0.278 |
| LNSC, µg/dL | 44% (46) | 46% (20) | 46% (20) | 38% (6) | 0.35 | 0.841 |
| 24-hour UFC, µg/24h | 34% (35) | 30% (13) | 41% (18) | 25% (4) | 1.91 | 0.386 |
| Inferior petrosal sampling | 38% (39) | 41% (18) | 27% (12) | 56% (9) | 4.58 | 0.101 |

**Supplemental Appendix Fig. 1. Standardized mean differences for baseline covariates before and after inverse probability of treatment weighting (IPTW)**

(A) Hispanic versus White non-Hispanic patients. (B) Black versus White non-Hispanic patients. Each row shows the absolute standardized mean difference (SMD) for one baseline covariate, before weighting (open circles) and after weighting (filled squares). Squares are filled in burgundy when residual imbalance exceeds the |SMD| > 0.20 threshold. The shaded band denotes the |SMD| ≤ 0.10 zone of adequate balance; dotted lines mark the |SMD| = 0.20 threshold. Pre-IPTW SMDs were calculated from the unweighted cohort; post-IPTW SMDs from the IPTW pseudo-population using stabilized weights truncated at the 1^st^-99th percentiles. The propensity model included age, sex, BMI, insurance type, distance to the surgical center, ADI national rank, comorbidity count, and Karnofsky Performance Status.

*^1^ bmi = body mass index; osa = obstructive sleep apnea; kps = karnofsky performance status; adi = area deprivation index; acth = adrenocorticotropic hormone; lnsc = late-night salivary cortisol; ufc = urinary free cortisol; mri = magnetic resonance imaging; iptw = inverse probability of treatment weighting; smd = standardized mean difference.*

**Supplemental Appendix Table 3. Performance metrics for the multinomial propensity score model used for IPTW**

Goodness-of-fit and discrimination metrics for the multinomial logistic regression propensity score model used to generate IPTW weights.

*Model included age, sex, BMI, insurance type, distance to the surgical center, ADI national rank, comorbidity count, and Karnofsky Performance Status. AUC reported as macro-averaged one-versus-rest. ^1^ auc = area under the receiver operating characteristic curve; iptw = inverse probability of treatment weighting.*

| **Metric** | **Value** |
| --- | --- |
| McFadden pseudo-R² | 0.204 |
| Nagelkerke pseudo-R² | 0.388 |
| Cox-Snell pseudo-R² | 0.337 |
| Classification accuracy | 65.7% |
| AUC (macro one-vs-rest) | 0.783 |

**Supplemental Appendix Table 3. Standardized mean differences for baseline covariates after inverse probability of treatment weighting (IPTW)**

Pairwise absolute standardized mean differences (aSMDs) between groups in the IPTW pseudo-population, comparing Hispanic vs. White non-Hispanic and Black vs. White non-Hispanic patients across all baseline covariates included in the propensity score model and additional clinical, radiographic, and endocrinologic characteristics.

*Balance thresholds: |SMD| < 0.10 (adequate), 0.10-0.20 (acceptable), and > 0.20 (residual imbalance). *0.10 < |SMD| ≤ 0.20; **|SMD| > 0.20. The multinomial propensity score model included age, sex, BMI, insurance type, distance to the surgical center, ADI national rank, comorbidity count, and Karnofsky Performance Status. Stabilized weights were truncated at the 1^st^-99th percentiles. Pre-IPTW and post-IPTW SMDs are visualized graphically in Supplemental Appendix Fig. 1. ^1^ smd = standardized mean difference; ; iptw = inverse probability of treatment weighting; bmi = body mass index; kps = karnofsky performance status; adi = area deprivation index; acth = adrenocorticotropic hormone; lnsc = late-night salivary cortisol; ufc = urinary free cortisol; cavernous sinus invasion.*

| Characteristic | SMD after IPTW | |
| --- | --- | --- |
|  | **Hispanic** | **Black** |
| Comorbidities and function | | |
| Age at surgery (years) | -0.01 | 0.02 |
| Male sex | -0.09 | 0.09 |
| BMI (kg/m²) | 0.13* | 0.36** |
| Hypertension | 0.31** | 0.60** |
| Diabetes mellitus | -0.01 | 0.56** |
| Obstructive sleep apnea | -0.15* | -0.39** |
| Obesity | 0.10 | -0.05 |
| Preoperative KPS | 0.13* | 0.24* |
| Access to care | | |
| Private insurance | -0.09 | -0.33** |
| Public insurance | 0.13* | 0.57** |
| Uninsured | -0.04 | -0.47** |
| ADI national rank | -0.25** | 0.02 |
| Distance to center (miles) | -0.20* | -0.01 |
| Presenting symptoms | | |
| Headache | -0.08 | 0.66** |
| Visual symptoms | 0.04 | -0.07 |
| Cognitive disturbance | 0.03 | -0.43** |
| Endocrinologic presentation | | |
| Serum cortisol (µg/dL) | -0.12* | 0.79** |
| Plasma ACTH (pg/mL) | -0.33** | 0.80** |
| LNSC (µg/dL) | -0.87** | -0.37** |
| 24-hour UFC (µg/24h) | -0.14* | 0.55** |
| Radiographic presentation | | |
| Tumor volume (cm³) | 0.12* | 0.09 |
| Macroadenoma | -0.04 | 0.19* |
| Occult tumor | -0.04 | -0.47** |
| CSI | 0.22* | -0.32** |
| Enhancement | 0.14 | 0.65** |
| Chiasmal compression | 0.15 | 0.44** |
| Tumor morphology | -0.17* | -0.88** |
| *Solid* | 0.00 | 0.84** |
| *Cystic* | -0.23* | -0.70** |
| *Mixed* | 0.11* | -0.39** |
